# Antibiotic Activity of a *Paraphaeosphaeria sporulosa*-Produced Diketopiperazine against *Salmonella enterica*

**DOI:** 10.3390/jof6020083

**Published:** 2020-06-10

**Authors:** Raffaele Carrieri, Giorgia Borriello, Giulio Piccirillo, Ernesto Lahoz, Roberto Sorrentino, Michele Cermola, Sergio Bolletti Censi, Laura Grauso, Alfonso Mangoni, Francesco Vinale

**Affiliations:** 1 Consiglio per la ricerca in agricoltura e l’economia agraria, Cerealicoltura e Colture Industriali. Via Torrino, 2; I-81100 Caserta, Italy; raffaele.carrieri@unina.it (R.C.); giuliopiccirillo@gmail.com (G.P.); ernesto.lahoz@crea.gov.it (E.L.); roberto.sorrentino@crea.gov.it (R.S.); michele.cermola@crea.gov.it (M.C.); 2 Istituto Zooprofilattico Sperimentale del Mezzogiorno, Via Salute, 2; Portici, 80055 Napoli, Italy; giorgia.borriello@cert.izsmportici.it; 3 Cosvitec, scarl, Via G. Ferraris, 171, 80100 Napoli, Italy; sergio.bolletti@cosvitec.ue; 4 Dipartimento di Agraria, Università degli Studi di Napoli Federico II. Via Università, 100; Portici, 80055 Napoli, Italy; laura.grauso@unina.it; 5 Dipartimento di Farmacia, Università degli Studi di Napoli Federico II. Via Domenico Montesano, 49; 80131 Napoli, Italy; alfonso.mangoni@unina.it; 6 Dipartimento di Medicina Veterinaria e Produzioni Animali—Università degli Studi di Napoli Federico II. Via Federico Delpino, 1; 80137 Napoli, Italy; 7 Consiglio Nazionale delle Ricerche, Istituto per la Protezione Sostenibile delle Piante, Via Università, 133, Portici, 80131 Napoli, Italy

**Keywords:** bioactive compounds, *Salmonella*, multidrug resistance, diketopiperazines, *Paraphaeophaeria sporulosa*, *cyclo*(L-Pro-L-Phe), secondary metabolites, endophytic fungus

## Abstract

A diketopiperazine has been purified from a culture filtrate of the endophytic fungus *Paraphaeosphaeria sporulosa*, isolated from healthy tissues of strawberry plants in a survey of microbes as sources of anti-bacterial metabolites. Its structure has been determined by nuclear magnetic resonance (NMR) and liquid chromatography–mass spectrometry (LC–MS) analyses and was found to be identical to *cyclo*(L-Pro-L-Phe) purified from species of other fungal genera. This secondary metabolite has been selected following bioguided-assay fractionation against two strains of *Salmonella enterica*, the causal agent of bovine gastroenteritis. The diketopiperazine *cyclo*(L-Pro-L-Phe), isolated for the first time from *Paraphaeosphaeria* species, showed minimum inhibitory concentration (MIC) values of 71.3 and 78.6 μg/mL against the two *S.* *enterica* strains. This finding may be significant in limiting the use of synthetic antibiotics in animal husbandry and reducing the emergence of bacterial multidrug resistance. Further in vivo experiments of *P. sporulosa* diketopiperazines are important for the future application of these metabolites.

## 1. Introduction

Animal infections caused by pathogenic bacteria are commonly treated with antibiotics. However, their massive and often improper use has led to a noticeable increase in microbial multidrug-resistance (MDR), considered a severe problem for global public health [1,2]. Antimicrobial resistance extends treatments and increases the risk of spreading the resistant microorganisms and an increase of health-care costs. In addition, the development of new antibiotics is restricted by economic and regulatory obstacles [3].

Overuse of drugs is also reported in animal husbandry, where antibiotics are used to prevent infection and as growth supplements [4,5]. In this field, previous reports showed that chronic treatments with a single drug can lead to MDR [6]. Many isolates belonging to the genus *Salmonella*—very common in cattle and considered one of the most relevant foodborne zoonoses—are resistant to multiple antibiotics [7,8]. The consumption of contaminated food of animal origin (eggs, meat, milk) may transfer resistant bacteria to humans, predisposing them for serious infections [8].

A very high number of new bioactive secondary metabolites have been reported in the literature in the last 20 years [9]. They are largely used in different areas, such as medicine, animal husbandry, environment and agriculture. Natural compounds are major sources of new active principles for drug formulates. All around the world, researchers investigate the biological properties of metabolites extracted mainly from plants, fungi and marine-derived organisms [10]. The number of bioactive molecules isolated from fungal species has increased since the early 1990s [9].

Endophytic fungi may colonize living plant tissue without causing any negative effects and are an important source of biologically active secondary metabolites. The molecules isolated from these fungi belong to different classes of natural compounds: isoprenoids, polyketides, amino acid derivatives, terpenoids, steroids, xanthones, chinones, phenols, isocumarines, benzopyranones, tetralones, cytochalasines and enniatines [11].

During a survey on fungi as a source of anti-bacterial metabolites, an endophytic fungus was isolated from the stem and crown of healthy strawberry plants. The objectives of the present study were to: (i) fully characterize this fungal isolate at the morphological and molecular levels; (ii) test the activity of the fungal culture filtrate and its organic extracts against two strains of *S. enterica*, the causal agent of bovine gastroenteritis; (iii) determine the half maximal inhibitory concentration (IC50) and minimum inhibitory concentration (MIC) of the resulting active fraction(s); and (iv) identify the bio-active compound(s) present in the active fraction(s).

## 2. Materials and Methods

### 2.1. Fungal Isolation and Morphological Characterization

Fungal isolations were performed from tissues (stem and crown) of healthy strawberry plants (*Fragaria x ananassa*) collected in April 2014 from various orchards located in Caserta province, Southern Italy. Tissue fragments (3–4 mm^2^) were excised with a surgical blade, dipped in 2% (vol/vol) NaClO for 30 s, rinsed twice in sterile distilled water and dried on sterile Whatman filter paper under a laminar flow hood. The samples were placed in 90 mm-diameter Petri dishes containing Potato Dextrose Agar (PDA, Conda, Madrid, Spain), amended with streptomycin sulphate (100 mg/L). The dishes were kept at 24 ± 2 °C in the dark. After 4–5 days of incubation, the fungal colonies emerging on the vegetable fragments were transferred on fresh PDA plates. Single-spore cultures were obtained by spreading 0.2 mL of a suspension containing 1 × 10^3^ conidia/mL, prepared from 7 days-old plates, on 1% water agar plates. Then, the germinated spores were transferred onto PDA dishes. The growing colonies were observed with an optical microscope to allow for a morphological characterization (colony diameter, colony color and degree of sporulation) (Olympus BH-2, Olympus America, Lake Success, NY, USA).

### 2.2. Molecular Characterization and Phylogenetic Analysis

The internal transcribed spacers (ITS) regions and the 5.8S gene, in the cluster of ribosomal genes, were sequenced for molecular characterization. Genomic DNA was extracted in triplicate with the DNeasy Plant Mini Kit (Qiagen, Hilden, Germany). Subsequently, an aliquot was PCR-amplified by using primers ITS4/ITS5 [12]. The reaction mixture contained: the DNA template (50 ng), 1× reaction buffer, 2 mM MgCl_2_, 200 µM of each dNTP, 400 nM of each primer and 2.5 U Taq DNA polymerase. PCR was performed in 38 cycles; each cycle consisted of three steps—denaturation at 95 °C (1 min), annealing at 55 °C (1 min) and extension at 72 °C (1 min)—followed by a final extension at 72 °C (10 min). Amplicons of the expected size were purified from agarose gel with the DNA Clean & Concentrator kit (Zymo Research, Orange, CA, USA), sequenced at Microgem (Naples, Italy) and then analyzed using the NCBI-BLASTn program [13]. The sequence of the isolate (named CP-1) was deposited in GenBank under the accession number MN017716. The phylogenetic analysis was performed using the molecular evolutionary genetics analysis (MEGA) software, version 6.0, including the sequences from *Paraphaeophaeria* spp. showing the highest similarity with the Italian isolate in the BLAST-generated alignment. The phylogenetic tree was constructed with the maximum likelihood methods based on the Tamura–Nei model using default parameters, and the statistical reliability of the branches was evaluated by bootstrapping (1000 replicates).

### 2.3. Liquid Culture and Metabolite Production

For the secondary metabolites production, CP-1 isolate was grown on PDA Petri dishes for a week at 24 °C in the dark. Five-mm diameter plugs were taken from actively growing margins of cultures and placed in Erlenmeyer flasks containing 5 L of potato dextrose broth (PDB, Conda, Madrid, Spain). The stationary cultures were incubated for 21 days at 24 °C in the dark. The fungal biomass was eliminated by filtration, first through sterile gauzes and then through 0.45-µm Whatman filters on vacuum. An aliquot (100 mL) of the filtered broth was extracted with an equal volume of ethyl acetate (EtAc) to evaluate the antibiotic activity.

### 2.4. Antibiotic Assay of Organic Extract

The organic extract was solubilized in ethanol (20 µg/µL) and tested against two *S. enterica* strains (S1 and S2), provided by the Istituto Zooprofilattico Sperimentale del Mezzogiorno (Portici, Naples, Italy). Five-mm diameter disks of Whatman paper were treated with 20 µL of extract and placed, after ethanol evaporation, on Luria broth agar (LBA, Conda, Madrid, Spain) Petri dishes previously inoculated with 200 µL of a bacterial suspension (5 × 10^6^ CFU/mL). Dishes treated only with 20 µL of solvent were used as negative control. Four paper disks were placed on each plate. Three dishes per essay were incubated at 37 °C for 24 h, and then the diameters of inhibition halos were measured.

### 2.5. Extraction and Isolation of Secondary Metabolites

The CP-1 culture filtrate (5 L) was acidified to pH 4 with 5 M HCl and extracted exhaustively with EtAc. The resulting organic extract was dried with Na_2_SO_4_ anhydrous, and the solvent was removed under reduced pressure at 40 °C (Rotavapor RV 10 IKA^®^ - Werke GmbH & Co. KG, Staufen, Germany). The residue was recovered and subjected to fractionation by direct phase column chromatography (silica gel; 200 g), using an elution gradient composed of dichloromethane (CH_2_Cl_2_)/methanol (MeOH) at different percentages (*v/v*). The collected fractions were then subjected to preparative thin layer chromatography (TLC), using the following solvents: CH_2_Cl_2_/MeOH, 90:10 (*v/v*), and combined into a total of six fractions (1–6). Among these, fraction number 2, biologically active against both *Salmonella* strains, was subjected to a further column chromatography, isocratically eluted with chloroform (CHCl_3_)/MeOH, 95:5 (*v/v*). The fractions thus collected were subjected to TLC (as described above) and combined into a total of eight sub-fractions.

Sub-fraction BC2, active against the *Salmonella* strains, was purified by semi-preparative high-performance liquid chromatography (HPLC-Agilent Technologies, Santa Clara, CA, USA). Separations were performed on a Luna C-18 column (250 × 10 mm, 10 μm, Phenomenex Torrance, CA, USA) eluted isocratically with a mixture of 30% MeOH and 70% H_2_O, using a flow of 5 mL/min and refractive index (RI) detection. Separations yielded a major peak with a retention time at 27.6 min, and then resulted in being pure compound **1** by nuclear magnetic resonance (NMR) and liquid chromatography–mass spectrometry (LC-MS).

### 2.6. LC–MS Analysis

Analyses were performed on an HPLC 1260 Infinity Series (Agilent Technologies, Santa Clara, CA, USA) equipped with a DAD (Diode Array Detector) system (Agilent Technologies) and coupled to a quadrupole–time of flight (Q-TOF) mass spectrometer, model G6540B (Agilent Technologies) with a Dual ESI source (Agilent Technologies). An Ascentis^®^ Express C-18 column (2.7 μm, 50 mm × 3.0 mm i.d., Supelco^©^, Bellefonte, PA, USA), held at a constant temperature of 37 °C, was used for separations. The mobile phase consisted of A: 0.1% (*v/v*) formic acid (FA) in water (H_2_O) and B: 0.1% formic acid (FA) in acetonitrile (ACN). Elution was done at a flow rate of 0.4 mL/min, and the gradient was as follows: starting condition 5% B ramping to 100% B until 6 min, held at 100% B for 2 min, lowering to 5% B in 2 min, and held at 5% B for 2 min as the equilibration time. The injection volume was 7 µL. UV spectra were collected by DAD, setting the detection wavelength at 210, 250 and 280 nm. Both chromatographic and spectral parameters were set using the Agilent MassHunter Data Acquisition Software, rev. B.05.01 (Agilent Technologies). The system operated in positive ion mode, and MS spectra were recorded in the *m/z* 50–1700 range as centroid spectra, with a speed of 3.3 spectra/s. The capillary was maintained at 2000 V, fragmentor voltage at 180 V, cone 1 (skimmer 1) at 45 V and Oct RFV at 750 V. The gas flow rate was set at 11 L/min, at 350 °C, and the nebulizer was set at 45 psig. A standard solution was infused by using an isocratic pump (1260 Infinity Series, Agilent Technologies) in order to perform the real-time lock mass correction. The solution consisted of two reference mass compounds: purine (C_5_H_4_N_4_ at *m/z* 121.050873, 10 µmol/L) and hexakis (1H,1H, 3H-tetrafluoropentoxy)-phosphazene (C_18_H_18_O_6_N_3_P_3_F_24_ at *m/z* 922.009798, 2 µmol/L). The flow rate was set at 0.06 mL/min, while the detection window and the minimum height were set at 1000 ppm and 10,000 counts, respectively, for the reference mass correction. LC–MS data were evaluated using MassHunter Qualitative Analysis Software B.06.00 and compared to known compounds included in an in-house database. Positive identifications were reported if the compound was detected with a mass error below 10 ppm and with a sufficient score. The high-resolution mass spectrum (HR-MS) was acquired in positive ion detection mode in the range of *m*/*z* 100–2000 with a resolution set to 60,000. The HR-ESI-MS experiment was performed on a Thermo LTQ Orbitrap XL mass spectrometer (Thermo Fisher Scientific Spa, Rodano, Italy).

### 2.7. Antibiotic Assay on Fractions and Pure Compound

The biological activity of the six fractions obtained with the first chromatographic separations (Section 2.5) were tested at 1 µg/µL using the disk method (Section 2.4). The agar dilution method was used to test the eight sub-fractions, subsequently obtained by the active fraction (n. 2). The tested concentrations were: 5, 9.5, 19 and 38 µg/mL.

The final concentrations were obtained by diluting the proper amount of the active sub-fraction (dissolved in ethanol) in agar cooled at 45 °C. The same quantity of amended agar per dish was allowed to solidify in 9-cm Petri dishes. The inoculum of the *Salmonella* strains was obtained by recovering bacterial cells from plates inoculated 24 h before and preparing a suspension in a sterile NaCl solution (0.9% *w/v*). The turbidity of the bacterial suspension was adjusted spectrophotometrically to 0.10 at 625 nm. The content of the colony-forming units was measured by a haematocytometer and confirmed to be 3 × 10^8^ CFU/mL. After a dilution to about 3 × 10^5^ CFU/mL, 10 µL of the suspension were added to each plate. Four replicates per sub-fraction concentration and strain were used. The negative controls consisted in Petri dishes containing only the solvent. The plates were incubated at 37 °C for 24 h, and the number of developed colonies was counted. The effect of the active sub-fractions was measured as the percent reduction of colonies compared to the control. To determine the IC and MIC values, the inhibition scores were fitted with the monomolecular growth function, also known as Mitscherlisch or von Bertalanffy law, with the following parameters:

% inhibition = c + (d − c) * (1 − exp(−dose/e))(1)

where c is the lower limit at zero dose, d is the upper asymptote and e is a parameter correlated with the slope. The analysis was done with the R environment [14] and the packages drc [15] and tidyverse [16].

## 3. Results

### 3.1. Morphological and Molecular Characterization of the Fungal Isolate

The fungus, isolated from the tissues of healthy strawberry plants, showed pycnidia, single or eustromatic and more complex, globose, superficial or immersed in the agar, glabrous, 130–280 µm diameter, initially pale and later dark, brown or black. Conidia, released through one, rarely two, ostioli, were subglobose, ellipsoid or pyriform, initially hyaline, then olivaceous-brown, 0 septate, 3.5–5 × 3–4 µm. Colonies on PDA reached a 32–38 mm diameter in 10 days, with an even to slightly ruffled, colorless to buff margin. Immersed mycelium appeared olivaceous in the center, covered by a dense, woolly mat, white to rosy or ochreous aerial mycelium. The reverse side was mostly pale luteous to ochreous, with darker areas in the center. Based on these morphological features, the fungus was ascribed to the *Paraphaeosphaeria sporulosa* species [17].

The amplification of 18S rDNA with ITS4 and ITS5 primers was successfully performed on five fungal isolates, identified by morphological observations. The five ITS sequences (482 bp in length) showed 100% identity to each other. The ITS sequence of the CP-1 isolate was deposited in GenBank under the accession number MN017716.1. By means of the NCBI BLAST algorithm, we found that the ITS sequence shared 99.79% nucleotide identity to the corresponding sequences of several *P. sporulosa* isolates.

**Figure 1:**
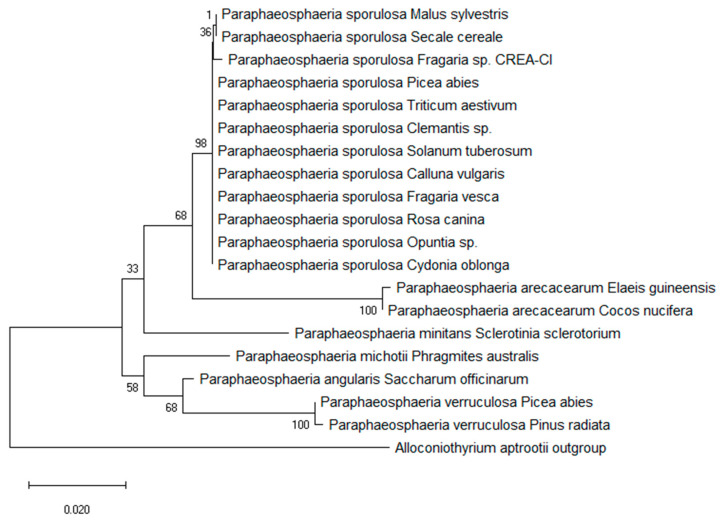
Phylogenetic analysis. Unrooted phylogenetic tree calculated from partial ITS regions (ITS1, 5.8S gene and ITS2) of rDNA of *Paraphaeosphaeria* species. *Paraphaeosphaeria sporulosa* Fragaria sp. CREA-CI indicates the CP-1 isolate obtained by the authors. The *Alloconiotirium aptrootii* sequence was used as the outgroup.

A phylogenetic tree was constructed using the rDNA sequence of our isolate and eighteen sequences of related species of *Paraphaeosphaeria* isolated from plants; the *Alloconiotirium aptrootii* sequence was used as the outgroup. *Paraphaeosphaeria sporulosa* isolated from strawberry plants clustered in a monophyletic group, supported by the bootstrap (98%) (Figure 1). *Paraphaeosphaeria arecacearum* resulted as being the closest species to *P. sporulosa*, while *P. verruculosa* resulted as being the most distant one. The bootstrap values are shown on the major branches of the phylogenetic tree to indicate the confidence level of each branch.

### 3.2. Isolation and Chemical Characterization of Bioactive Compound ***1***

The extraction with EtAc of 5 L of culture filtrates yielded 550 mg of crude extract, which was further subjected to fractionation by direct phase column chromatography and to preparative TLCs when needed. A second purification led to a total of eight sub-fractions, named BC1, BC2, DE1, DE2, DE3, F1, F2 and F3. Among these, only sub-fraction BC2 was active against *Salmonella* strains and was subjected to further purification and chemical characterization.

**Figure 2:**
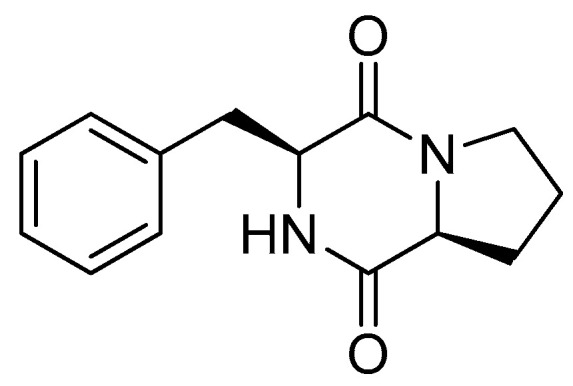
Chemical structure of 1, *cyclo*(L-Pro-L-Phe) isolated from *Paraphaeosphaeria sporulosa*.

From this sub-fraction, the semi-preparative HPLC separations yielded a major peak with a retention time at 27.6 min, which resulted in being a pure compound by nuclear magnetic resonance (NMR, Figure S1) and liquid chromatography–mass spectrometry (LC–MS, Figure S2)) analyses (**1**, 5.3 mg, Figure 2). The high-resolution, electrospray-ionization mass spectrum (HR–ESI–MS, Figure S3) of compound **1** displayed an [M + H]^+^ ion peak at *m/z* 245.1286, corresponding to the molecular formula C_14_H_16_N_2_O_2_. The molecular formula and analysis of the ^1^H NMR data of compound **1** suggested a diketopiperazine (cyclic dipeptide) structure. The presence in the spectrum of five aromatic protons between δ 7.47 and 7.28 suggested a mono-substituted benzene, and therefore a Phe residue, while the absence of any methyl resonance and heteroatoms, other than those involved in the two peptidic bonds, pointed to a proline residue. Indeed, the ^1^H NMR spectrum of compound **1** matched perfectly the spectrum reported for *cyclo*(L-Pro-L-Phe) [18,19], and this also established the relative configuration (either L,L or D,D) of the two amino-acid residues. The L,L absolute configuration of the molecule was deduced by the negative value measured for its optical rotation [α]_D_ = −91 (c = 0.15, EtOH), in accordance with the [α]_D_ = −101.62 reported for *cyclo*(L-Pro-L-Phe) (Figure 2) [19].

### 3.3. Antibiotic Activity

**Figure 3:**
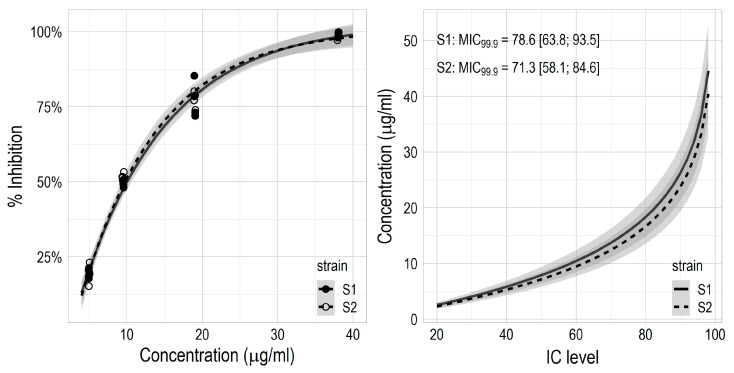
Inhibition of two *Salmonella enterica* strains (S1 and S2) using different concentrations of **1** (**left**) and IC50 levels and MICs (**right**).

The culture filtrate of *P. sporulosa* and its organic extracts showed evident inhibition halos on Petri plate assays against both *S. enterica* strains (Figure S5). The bioassay-guided fraction yielded one main compound responsible for the detected activity (Figure S5), fully characterized as **1**, *cyclo*(L-Pro-L-Phe). This secondary metabolite was used to perform the agar-dilution assay. The effect of **1** was similar on both *S. enterica* strains, with the estimated curves of inhibition vs. concentration largely overlapping, and rather strong, with a complete inhibition at the highest tested dose of 38 μg/mL (Figure 3).

The inhibition response was comparable between the two *Salmonella* strains. However, the S2 strain showed a slightly higher sensitivity compared to the S1 strain at intermediate and higher concentrations of **1**: average IC50 7.9 against 7.2 μg/mL; average MIC_99.9_ 71.3 against 78.6 μg/mL (Figure 3).

## 4. Discussion

Diketopiperazines are cyclic dipeptides obtained by the condensation of two α-amino acids, biosynthesized by different organisms and considered very interesting for drug discovery [20]. Due to their structure (heterocyclic system), these secondary metabolites can bind several receptors, resulting in a wide range of biological properties, including antimicrobial, antitumor and anti-hyperglycemic activities [21]. Antibiosis against bacteria such as *Bacillus subtilis*, *Staphylococcus aureus*, *Escherichia coli* and *Pseudomonas aeruginosa* has been reported [22].

Diketopiperazines are produced by several endophytic fungi, including *Colletotrichum gloeosporioides*, *Fusarium* spp. and *Penicillium crustosum* [23]. Endophytic fungi, residing in the tissues of healthy plants, and marine-derived fungal species are an unexplored source of bioactive secondary metabolites, with potential application in the agriculture, medicine and food industries [24,25].

*Paraphaeosphaeria* spp. are ubiquitous fungi isolated from soil and different plants [17,26]. *Paraphaeosphaeria sporulosa* (basionym *Coniothyrium sporulosum* or *Paraconiothyrium sporulosum*) is a cosmopolitan soil-borne fungus [27], frequently found as an endophyte in the roots and rhizomes of various plant species [28,29]. This fungus is known to produce various metabolites with different biological properties: sporulosaldeins, active against *Candida* spp. [30]; sporulosol, a compound with a not significant cytotoxicity against tumor cell lines [31]; and mycosporulone, a butanolide metabolite active against penicillin-resistant strains of *P. aeruginosa* and *S. aureus* [32].

To the best of our knowledge, this is the first study reporting: (i) *Fragaria* sp. as the host of the endophyte *P. sporulosa*; (ii) the isolation of a diketopiperazine from the culture filtrate of this fungus; (iii) the biological activity of a fungal diketopiperazine against *S. enterica*, the causal agent of bovine gastroenteritis; and (iv) the isolation of *cyclo* (L-Pro-L-Phe) from this fungus and the evaluation of its antibiotic activity.

## 5. Conclusions

The association between the use of antibiotics in animal production and anti-microbial resistance phenomena is very relevant among bacteria belonging to the genus *Salmonella* [33]. The serious problem of MDR motivates the use of novel antibiotics based on bioactive metabolites of fungal origin. Therefore, the diketopiperazine isolated from *P. sporulosa* could represent a promising tool to limit the use of marketed antibiotics in animal husbandry. Further investigations should be conducted on the biological properties of this compound with in vivo experiments.

## Supplementary Materials

The following are available online at https://www.mdpi.com/2309-608X/6/2/83/s1, Figure S1. ^1^H NMR spectrum of *cyclo* (L-Pro-L-Phe). Figure S2. LC-MS qTOF spectrum of *cyclo* (L-Pro-L-Phe). Figure S3. High resolution electrospray mass spectrum of *cyclo* (L-Pro-L-Phe). Figure S4. *Paraphaeosphaeria sporulosa* CREA-CI grown on potato dextrose agar (A); (B) and (C), microscopic pictures of conidia. Figure S5. Inhibition halos on Petri plates of *Paraphaeosphaeria sporulosa* CREA-CI extract on *Salmonella enterica* strains (A); inhibition halos on Petri plates of *cyclo* (L-Pro-L-Phe) on *Salmonella enterica* strains (B—right halos).
