# Supplementary material for: Antibiotic Activity of a Paraphaeosphaeria sporulosa-Produced Diketopiperazine against Salmonella enterica
Source: J Fungi (Basel). 2020 Jun 10;6(2):83. doi: 10.3390/jof6020083 (PMC7344678; doi:10.3390/jof6020083)
Supplement: Supplementary file 1 [file jof-06-00083-s001.pdf]

# SUPPLEMENTARY MATERIAL

**Figure S1.** $^{1}H$ NMR spectrum of *cyclo*(L-Pro-L-Phe) (500 MHz, CDCl3).
**Figure S2.** LC-MS qTOF spectrum of *cyclo*(L-Pro-L-Phe).
**Figure S3.** High resolution electrospray mass spectrum of *cyclo*(L-Pro-L-Phe).
**Figure S4.** *Paraphaeosphaeria sporulosa* CREA-CI grown on potato dextrose agar (**A**); (**B**) and (**C**), microscopic pictures of conidia.
**Figure S5.** Inhibition halos on Petri plates of *Paraphaeosphaeria sporulosa* CREA-CI extract on *Salmonella enterica* strains (A); inhibition halos on Petri plates of *cyclo*(L-Pro-L-Phe) on *Salmonella enterica* strains (B – right halos).

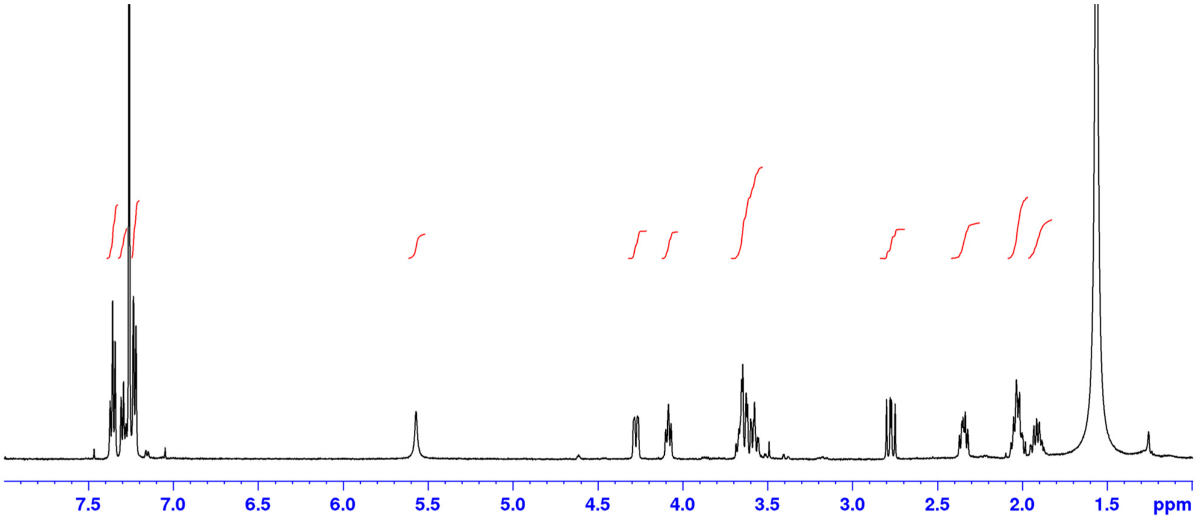


**Figure S1.** $^1$H NMR spectrum of *cyclo*(L-Pro-L-Phe) (500 MHz, $CDCl_3$).

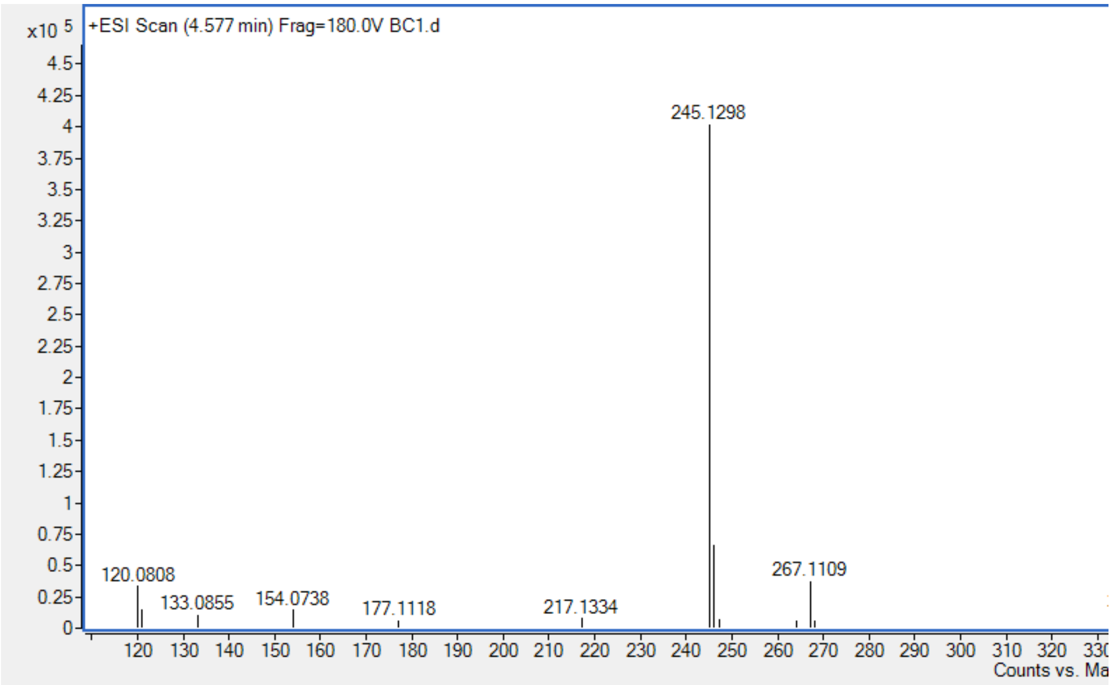


**Figure S2.** LC-MS qTOF spectrum of *cyclo*(L-Pro-L-Phe) (recorded on a qTOF instrument).

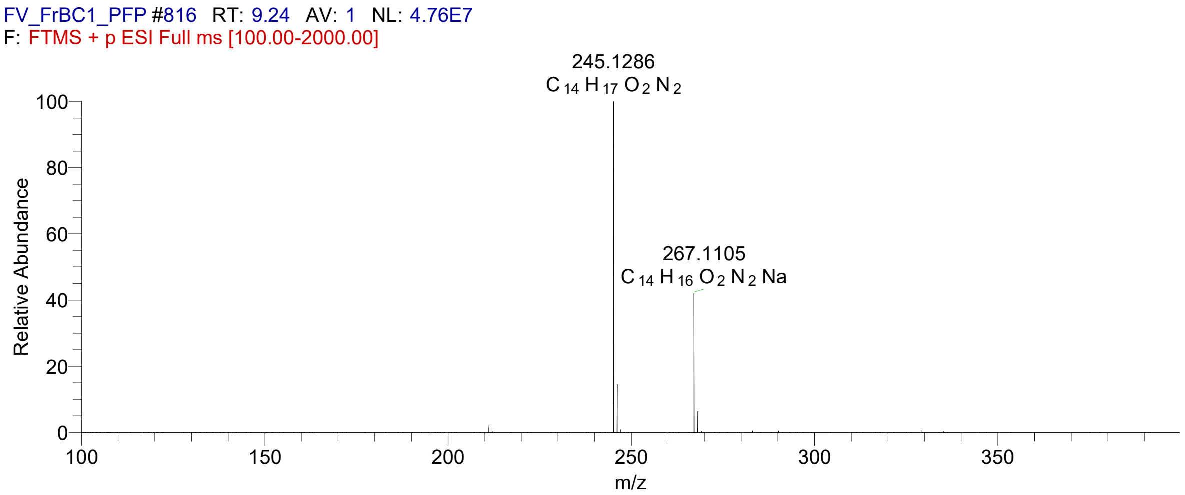


**Figure S3.** High resolution electrospray mass spectrum of *cyclo*(L-Pro-L-Phe) (recorded on an LTQ Orbitrap XL instrument).

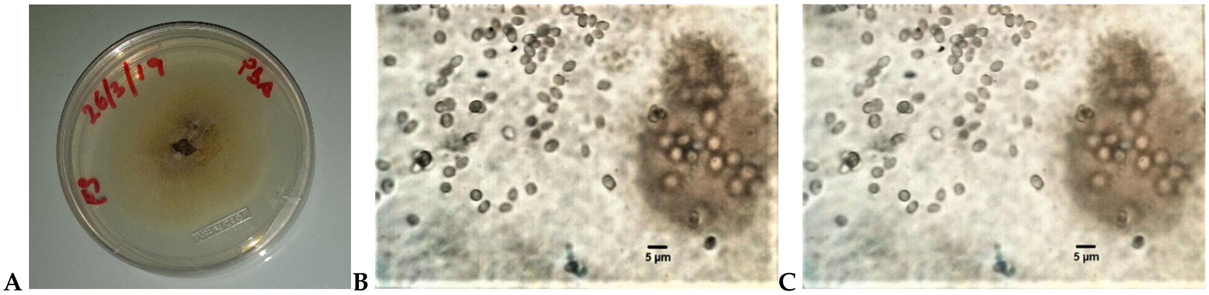


**Figure S4.** *Paraphaeosphaeria sporulosa* CREA-CI grown on potato dextrose agar (**A**); (**B**) and (**C**), microscopic pictures of conidia.

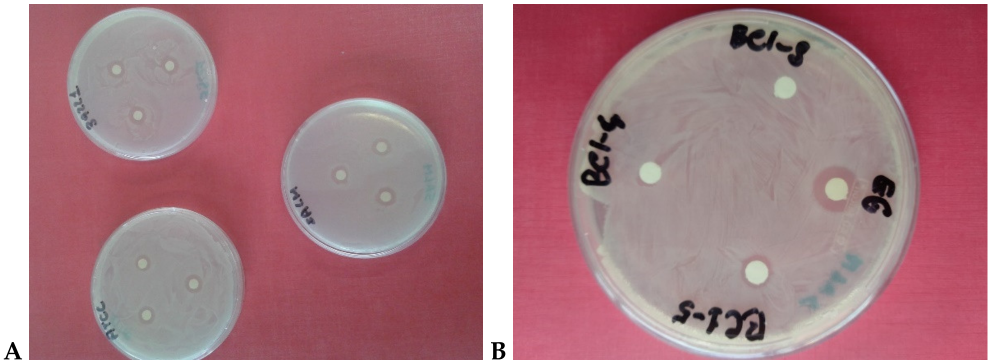


**Figure S5.** Inhibition halos on Petri plates of *Paraphaeosphaeria sporulosa* CREA-CI extract on *Salmonella enterica* strains (A); inhibition halo on Petri plates of *cyclo*(L-Pro-L-Phe) on *Salmonella enterica* strain (B – right halo).
